# Editorial Notes

**Published:** 1937

**Authors:** 


### Editorial Notes

**Amalgamation of the B.R.I. and B.G.H.**

THE Governors of the Royal Infirmary and of the General Hospital have, at special meetings held on February 23rd and March 15th respectively, passed a resolution approving the amalgamation of these two institutions. The resolution authorizes the two Committees of Management to proceed with plans for carrying the amalgamation into effect, so that there shall be central administration, a pooling of funds and finances, the formation of one joint honorary staff, and such centralization of functions as may be agreed upon. As the *Western Daily Press* observed on February 25th: "It is indicative of the change which opinion, both lay and professional, has undergone that no opposition was raised to this proposal" at the meetings of either body of Governors. As the writer of the leading article went on to remark: "Common sense and the logic of events are at last proving too strong for tradition, and with the coming into force of this amalgamation a new era in local hospital administration will open." Miss Hilda Wills (President), in submitting the resolution to the Governors of the General Hospital, put the reasons for amalgamation into a single apt sentence: "The onward march of scientific knowledge is too rapid and too imperious to admit of our medical institutions standing apart in isolated units any longer if Bristol is to retain its old and deservedly high reputation as a centre of medical knowledge and skill." The form which the amalgamation is to take will require careful and perhaps long deliberation. The Committees of the two institutions did not go to their Governors' Meetings with plans already prepared. To have made plans before receiving authority from the Governors might well have led to a rebuff.

Now, however, the Committees may go on to the development of schemes to implement the resolution. It is evident that for the present the institutions will continue as two general hospitals with little or no change in the facilities which they offer to patients. Two minor improvements had been agreed to before the amalgamation was decided upon, and these will come into effect at once.

A joint department for the treatment of fractures and a joint Radiological Department will be instituted in the near future. These measures of economy are independent of the amalgamation plan, and would have been introduced even if the two institutions had remained separate.

The first matter to be investigated is the pooling of financial resources and the devising of such a form of central administration as will command the approval of all present subscribers to both institutions and will appeal to the generosity of all well-wishers to the Voluntary System. It must appeal also to the subscribers to any contributory schemes which are now or may in the future be in existence.

From all sides expressions of satisfaction are heard, and it is clear that public opinion in Bristol looks upon the amalgamation idea with favour. We trust that the measures that will be necessary to give effect to the Governors' wishes will prove likewise acceptable.

**Bicentenary of the Royal Infirmary.**

JUST over two hundred years ago a plan for establishing an Infirmary in Bristol was conceived by Dr. Bonython.

That Bonython first mooted the project is clear from a letter of his written on December 11th, 1736, and quoted in Munro Smith's *History of the Bristol Royal Infirmary.* "For this last half year," he wrote, "I have been working hard at a scheme which, if I can bring it to bear, will make a very great difference in my way of living. It is to set up in this populous and rich city an Infirmary for sick and wounded by an annual subscription. . . . I have printed my proposals and opened our subscription book where we have some very good names." Actually the first minute book shows that "on the 22nd of November, 1736, a subscription was open'd for erecting an Infirmary in the City of Bristol."

The first general meeting of subscribers was summoned for the 23rd of December in that year.* The early rules of the Institution were drawn up by Sir Michael Foster, who had been made Recorder of Bristol in 1735. John Elbridge, Controller of His Majesty's Customs, was appointed Treasurer, and formally accepted office at the second board meeting on February 4th, 1736–7. In a pamphlet published in 1775 on *Bristol Hospitals, Alms Houses and Schools,* it is stated that the Bristol Infirmary was built and furnished at the sole expense of John Elbridge. The full extent of his generosity is not known, but he was a notable benefactor and well deserves his recognized

* In a recent issue of this *Journal* (1934, vol. li., p. 280) it was explained how the date 1735 came to be given as the year of the foundation of the Bristol Royal Infirmary.

place as the Founder of the Institution—even though Bonython may have been its first "planner."

On June 20th, 1737, the Infirmary was quietly opened for out-patients, but the ceremonial opening took place on December 13th, when the Mayor and Corporation, the Faculty and the Trustees met at the Infirmary, proceeded to Divine Service in St. James's Church, and concluded the day with a dinner at the Nag's Head Tavern.

It was on this day (December 13th, 1737) that the wards were formally opened and the first in-patients admitted. During the first year of the Infirmary's existence the number of patients admitted to the wards was 194, and the out-patients treated numbered 232, making a total of 426; the cost of maintaining the Institution was £433 16s., scarcely more than £1 per patient! In 1936 the number of in-patients was 10,305, that of out-patients 70,570, making a total of 80,875, the cost was £72,191 10s. 5d., less than £1 per patient.

From these figures it will be seen that the patients have increased approximately two-hundredfold, whilst the expenses have only multiplied one hundred and seventy times.

We are accustomed to regard an increase in the cost of living as inevitable; it appears as though with the extension of the Bristol Royal Infirmary and vast expansion of its work there has been no inconsiderable shrinking of real cost during the last two hundred years. Economists say that the pound of 1737 represented the purchasing power of three pounds in 1937. The managers of the Bristol Royal Infirmary to-day cannot be accused of extravagance.

The manner in which the Bicentenary will be celebrated has not been finally decided. A fête at the Zoological Gardens is announced to take place on July 7th, 8th, 9th and 10th. Other ceremonies and festivities are contemplated, the arrangements for which will be made known shortly.

It is hoped that at some date in October there will be a general re-union of staff, students and nurses, past and present.

**Colston Medical Research Fellowships.**

The Council of the Colston Research Society, Bristol, offers one or more Fellowships for 1937–38 of not less than £100 per annum for Research work primarily in Clinical subjects, though other medical subjects are not excluded. The work must be carried out in the University of Bristol or in any of the Clinical Institutions associated with the University.

Awards will be made in June or July, 1937. Applications should be forwarded forthwith to the Hon. Secretary of the Colston Research Society, 12 Small Street, Bristol 1.

